# Supplementary material for: Flavored e-cigarettes modulate embryo development, fetal growth, and potentiate early fetal demise without nicotine
Source: Commun Med (Lond). 2025 Aug 28;5:373. doi: 10.1038/s43856-025-01094-0 (PMC12394398; doi:10.1038/s43856-025-01094-0)
Supplement: Supplementary file 2 — Supplemental Information [file 43856_2025_1094_MOESM2_ESM.pdf]

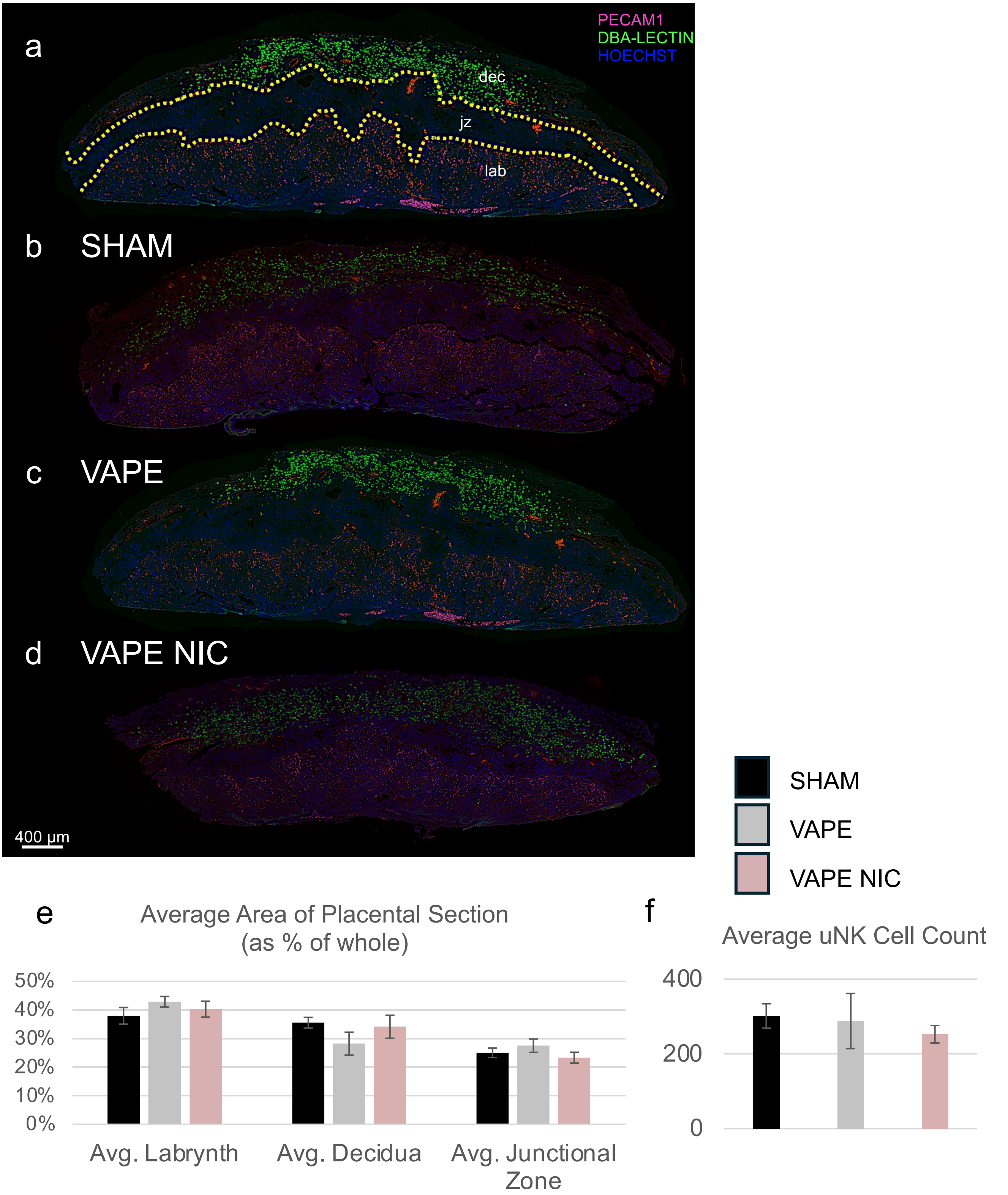

Supplementary Figure 1

Placenta Exposed with E-Cigarette Vapors Exhibit No Overt Morphology Changes.

Placentas were immunofluorescent stained with PECAM1 (pink stain), DBA-LECTIN (green stain), and HOECHST (blue stain) antibodies. Schematic describing the placental layers that were measured (dec=decidua, lab=labyrinth, jz=junctional zone) (a) and stained representative placentas per group (scale bar=400µm) (b-d). Area of placenta layers are graphed across exposure groups (e) (N=5). Uterine natural killer (uNK) cells were counted from e-cigarette exposed placentas (f) (N=5). SHAM depicted as black bars (e-f); e-cigarette vapors without nicotine (VAPE) depicted as grey bars (e-f); e-cigarette vapors with nicotine (VAPE NIC) depicted as pink bars (e-f). Error bars indicate mean ± SEM.

Primer	Sequence (5'-3')
18s_F	ATGCTCTTAGCTGAGTGTCCCG
18s_R	ATTCCTAGCTGCGGTATCCAGG
Ptgs2_F	TGAGCAACTATTCCAAACCAGC
Ptgs2_R	GCACGTAGTCTTCGATCACTATC
Hif1a_F	ACCTTCATCGGAAACTCCAAAG
Hif1a_R	CTGTTAGGCTGGGAAAAGTTAGG
Gpx2_F	GCCTCAAGTATGTCCGACCTG
Gpx2_R	GGAGAACGGGTCATCATAAGGG
Gpx3_F	CCTTTTAAGCAGTATGCAGGCA
Gpx3_R	GGGGAGTATCTCCGAGTTCTC
Mapk1_F	GGTTGTTCCCAAATGCTGACT
Mapk1_R	CAACTTCAATCCTCTTGTGAGGG
Txnrd1_F	GGGTCCTATGACTTCGACCTG
Txnrd1_R	AGTCGGTGTGACAAAATCCAAG

Supplementary Table 1

List of SYBR Primers Utilized in Gene Expression Studies.

Primers were queried from Harvard primer bank and validated before use in these studies.
