# Supplementary material for: Flavored e-cigarettes modulate embryo development, fetal growth, and potentiate early fetal demise without nicotine
Source: Commun Med (Lond). 2025 Aug 28;5:373. doi: 10.1038/s43856-025-01094-0 (PMC12394398; doi:10.1038/s43856-025-01094-0)
Supplement: Supplementary file 3 — Description of Additional Supplementary Files [file 43856_2025_1094_MOESM3_ESM.pdf]

Supplementary Data Legends

Supplementary Data 1

The “Figure a” tab includes raw data for litter number per dam corresponding to Figure 1a. The “Figure b” tab reports cotinine concentrations measured and displayed in Figure 1b. The “Figure f” tab displays erythrocyte accumulation scores across exposure groups corresponding to the graph in Figure 1f. The “Figure i” tab displays raw embryo elongation lengths measured across groups and graphed in Figure 1i. The “Figure j” tab reports the original embryo elongation rates in the “Figure i” tab with calculated averages. Difference from the respective group mean is calculated in the “Figure j” tab, displayed, and graphed in Figure 1j.

Supplementary Data 2

The “Figure a” tab displays values for average embryo weights per litter across exposure groups displayed in Figure 2a. The “Figure b” tab reports average placental weights per litter across groups displayed in Figure 2b. The “Figure c” tab displays average embryo to placental weight ratios per litter across groups, graphed in Figure 2c. The “Figure d” tab displays individual fetal to placental weight ratios reported in Figure 2d. Total resorptions observed per dam are displayed in the “Figure e” tab corresponding to Figure 2e. The “Figure f” tab reports the resorption distribution observed across groups, graphed in Figure 2f. The “Figure g” tab uses original data from the “Figure f” tab and calculates the resorption incidence per group across the total resorptions observed, displayed in Figure 2g.

Supplementary Data 3

The tabs correspond to the figures displayed in Figure 3 reporting the raw quantitative real-time reverse transcriptase polymerase chain reaction (qRT-RT-PCR) values for 18s and the corresponding reported gene. The ddCT method is used for the calculations and repeated for compiled and sex-specific results. The tab entitled “Figure a” reports data on hypoxia inducible factor 1, alpha subunit (*Hif1a*), displayed in Figure 3a. The tab “Figure b” reports the data for prostaglandin-endoperoxide synthase 2 (*Ptgs2*), displayed in Figure 3b. The “Figure c” tab reports data for glutathione peroxidase 2 (*Gpx2*), reported in Figure 3c. The “Figure d” tab reports data for glutathione peroxidase 3 (*Gpx3*), graphed in Figure 3d. The “Figure e” tab displays the data for thioredoxin reductase 1 (*Txnrd1*), reported in Figure 3e. The “Figure f” tab reports the data for mitogen-activated protein kinase 1 (*Mapk1*), graphed in Figure 3f.

Supplementary Data 4

The “Figure e” tab displays the placental area calculations across the placental layers in placentas from different exposure groups. Placental layer area percentage of the total placenta is calculated and averaged across groups. The average areas of the placental layers are graphed in Supplementary Figure 1e. The “Figure f” tab reports raw and averaged uterine natural killer (uNK) cell counts across exposure groups graphed in Supplementary Figure 1f.
