# Supplementary material for: Flavored e-cigarettes modulate embryo development, fetal growth, and potentiate early fetal demise without nicotine
Source: Commun Med (Lond). 2025 Aug 28;5:373. doi: 10.1038/s43856-025-01094-0 (PMC12394398; doi:10.1038/s43856-025-01094-0)
Supplement: Supplementary file 8 — Reporting Summary [file 43856_2025_1094_MOESM8_ESM.pdf]

nature portfolio

Corresponding author(s): Margeaux W. Marbrey

Last updated by author(s): Aug 3, 2025

Reporting Summary

Nature Portfolio wishes to improve the reproducibility of the work that we publish. This form provides structure for consistency and transparency in reporting. For further information on Nature Portfolio policies, see our Editorial Policies and the Editorial Policy Checklist.

Statistics

For all statistical analyses, confirm that the following items are present in the figure legend, table legend, main text, or Methods section.

n/a	Confirmed	
☐	☒	The exact sample size (*n*) for each experimental group/condition, given as a discrete number and unit of measurement
☐	☒	A statement on whether measurements were taken from distinct samples or whether the same sample was measured repeatedly
☐	☒	The statistical test(s) used AND whether they are one- or two-sided<br>*Only common tests should be described solely by name; describe more complex techniques in the Methods section.*
☐	☒	A description of all covariates tested
☐	☒	A description of any assumptions or corrections, such as tests of normality and adjustment for multiple comparisons
☐	☒	A full description of the statistical parameters including central tendency (e.g. means) or other basic estimates (e.g. regression coefficient) AND variation (e.g. standard deviation) or associated estimates of uncertainty (e.g. confidence intervals)
☐	☒	For null hypothesis testing, the test statistic (e.g. *F*, *t*, *r*) with confidence intervals, effect sizes, degrees of freedom and *P* value noted<br>*Give P values as exact values whenever suitable.*
☒	☐	For Bayesian analysis, information on the choice of priors and Markov chain Monte Carlo settings
☒	☐	For hierarchical and complex designs, identification of the appropriate level for tests and full reporting of outcomes
☒	☐	Estimates of effect sizes (e.g. Cohen's *d*, Pearson's *r*), indicating how they were calculated

Our web collection on statistics for biologists contains articles on many of the points above.

Software and code

Policy information about availability of computer code

Data collection	Data was tabulated using publicly available Microsoft Excel software.
Data analysis	Data was analyzed using publicly available GraphPad Prism software.

For manuscripts utilizing custom algorithms or software that are central to the research but not yet described in published literature, software must be made available to editors and reviewers. We strongly encourage code deposition in a community repository (e.g. GitHub). See the Nature Portfolio guidelines for submitting code & software for further information.

Data

Policy information about availability of data

All manuscripts must include a data availability statement. This statement should provide the following information, where applicable:

- Accession codes, unique identifiers, or web links for publicly available datasets
- A description of any restrictions on data availability
- For clinical datasets or third party data, please ensure that the statement adheres to our policy

All data that support the findings of this study are available within the paper and its Supplementary Information. Figure 1 (a,b,f,i,j) source data is described in Supplementary Data 1, Figure 2 (a-g) source data is found in Supplementary Data 2, and Figure 3 (a-f) source data is described in Supplementary Data 3. Each figure with corresponding source data is listed in the specified Supplementary Data file in a demarcated figure tab. Supplementary Figure 1 (e-f) source data is reported in

Supplementary Data 4. Primers used for gene expression studies are listed in Supplementary Table 1. All other data are available from the corresponding author upon reasonable request.

Research involving human participants, their data, or biological material

Policy information about studies with human participants or human data. See also policy information about sex, gender (identity/presentation), and sexual orientation and race, ethnicity and racism.

Reporting on sex and gender	No human research participants were incorporated in this study.
Reporting on race, ethnicity, or other socially relevant groupings	No human research participants were incorporated in this study.
Population characteristics	No human research participants were incorporated in this study.
Recruitment	No human research participants were incorporated in this study.
Ethics oversight	No human research participants were incorporated in this study.

Note that full information on the approval of the study protocol must also be provided in the manuscript.

Field-specific reporting

Please select the one below that is the best fit for your research. If you are not sure, read the appropriate sections before making your selection.

☒ Life sciences ☐ Behavioural & social sciences ☐ Ecological, evolutionary & environmental sciences

For a reference copy of the document with all sections, see nature.com/documents/nr-reporting-summary-flat.pdf

Life sciences study design

All studies must disclose on these points even when the disclosure is negative.

Sample size	Sample size was determined based on power analysis for expected measurable differences in weights, embryo lengths, placental areas, and molecular expression analyses performed with expertise in the field.
Data exclusions	GraphPad Prism outlier analysis or the Grubbs Test was performed on datasets with a p-value <=0.05 when applicable and appropriate. This analysis was used to identify and exclude outliers in the data. These scientifically founded exclusions resulted in a more refined understanding of the results and data observed in nature. Dams were also excluded from day 12.5 experiments if less than 2 viable embryos were present in the uterus.
Replication	The experiments were performed on three or more animals or tissues and often more than three dams, with embryos and placental pairs reaching up to 90. Thus, multiple replicate animals were used to determine these conclusions.
Randomization	Samples were analyzed and technically handled in an unbiased manner and were assessed in the same way regardless of treatment. Animals were separated by litter so littermates were exposed and compared across different treatment groups. Animals were randomly chosen and placed into treatment groups from originating litters. No computers were involved in randomization algorithms.
Blinding	As samples were labeled only with arbitrary numbers, the analyzer performed the analysis without knowledge of treatment and thus, without bias. Individuals performing the necropsy operated without bias as the mouse number is generic with no descriptors of treatment condition. Confounding variables were minimized by exposing animals in the same way, for the same amount of time, by the same technician when possible.

Reporting for specific materials, systems and methods

We require information from authors about some types of materials, experimental systems and methods used in many studies. Here, indicate whether each material, system or method listed is relevant to your study. If you are not sure if a list item applies to your research, read the appropriate section before selecting a response.

Materials & experimental systems

n/a	Involved in the study
☐	☒ Antibodies
☒	☐ Eukaryotic cell lines
☒	☐ Palaeontology and archaeology
☐	☒ Animals and other organisms
☒	☐ Clinical data
☒	☐ Dual use research of concern
☒	☐ Plants

Methods

n/a	Involved in the study
☒	☐ ChIP-seq
☒	☐ Flow cytometry
☒	☐ MRI-based neuroimaging

Antibodies

Antibodies used

PECAM1, Millipore Sigma MAB1398Z ; DBA-LECTIN, VWR #101097-972; Cy3-goat, anti-Armenian hamster VWR #102646-984; Hoechst Thomas Scientific #33258. All antibodies were performed according to manufacturer's instructions. Absent primary and absent secondary controls were used to confirm accurate staining.

Validation

These antibodies were all validated by the source manufacturer and have been previously used and published in these applications in murine placental tissues.

Animals and other research organisms

Policy information about studies involving animals; ARRIVE guidelines recommended for reporting animal research, and Sex and Gender in Research

Laboratory animals

C57BL/6J mice derived from Jackson Labs were used for these studies with an age >8 weeks. Only female mice were used for experiments as these studies centered on female reproduction and pregnancy.

Wild animals

This study did not incorporate wild animals.

Reporting on sex

Since the central focus of this manuscript was pregnancy, the majority of the experiments were performed on pregnant female mice as pregnancy is a condition limited to females. However, placental-fetal pairs were sexed and analyzed separately for RNA expression changes. Thus, male and female fetal sex was determined via genotyping described in the methods. Data analysis for placental RNA molecular changes were analyzed in a compiled form and also by sex.

Field-collected samples

This study did not involve samples caught in the field.

Ethics oversight

The Institution for Animal Care and Use Committee at the University of North Carolina Chapel Hill, NC approved the animal protocol implemented to complete these studies.

Note that full information on the approval of the study protocol must also be provided in the manuscript.

Plants

Seed stocks

n/a

Novel plant genotypes

n/a

Authentication

n/a
